# CONAA Council on Nursing & Anthropology Events

**DOI:** 10.1177/2333393619838883

**Published:** 2019-03-19

**Figure:**
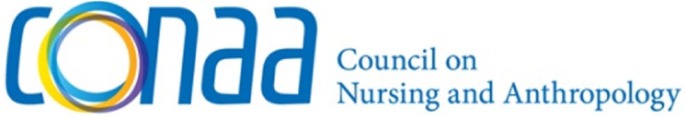


**CONAA Sessions and Papers**

**SfAA Portland Day Tuesday March 19**:

**All events are free and open to the public and local community members**.

**(T-64) TUESDAY 12:00-1:20**

**Broadway III**

***Portlandia: Myth or “Keep Portland Weird” Reality? (CONAA)***

CHAIRS: **BREDA, Karen** (U Hartford) and **PALUZZI, Jo** (Independent)

Can there be a unifying ethos within a diverse, sprawling, urban population? How might it shape socio-political identities in the current era? From its top spot in the microbrew universe to its award-winning vineyards, vibrant arts, indie music, seasonal festivals, and unique traditions (Naked Bike Ride, anyone?), Portland holds a special place in the national imagination. Our panel of Portlandians will share just how weird the P-City is (or not). Join us in the Rose City (aka Beervana, Rip City, Stumptown, Bridgetown) to explore the personality of this singular US city as we identify what unites Portlandians (or not).

**BULLOCK, Amanda** (Literary Arts) is the Director of Public Programs at the non-profit Literary Arts organization founded in 1984. With past experience in the publishing industry, Amanda now creates diverse and inclusive event experiences. Her organization supports writers and promotes writing and in doing so, creates and supports readers. Literary Arts has long had a robust presence in Portland high schools and sponsors or co-sponsors popular events such as the Portland Book Festival (formerly Wordstock), the Portland Lit Crawl (think of a literary pub-crawl), and the popular Portland Arts and Lectures series.

**CURRIER, Terry** (Music Millennium) Terry is the only member of the panel (so far) to have an official day named for him in Portland: August 12 is Terry Currier Day in the city. He is the owner of Portland’s oldest record store, Music Millennium (which this month celebrates their 50th anniversary). Terry also founded the Oregon Music Hall of Fame and has served on multiple local festival and music boards. Millennium Music is not just a store, it is an experience. Indie musicians are featured in live, in-store concerts; just one of many ways Terry provides support for local musicians.

**HEDGMON, Lee** (Ground Breaker Brewing, McMenamins Cornelius Pass Distillery, and Barreled Bee) Lee returned to Portland after completing graduate school in Minnesota. The year was 2010 and Portland’s reputation as the center of the indie brewing universe, now firmly established, was just beginning to take off. She is active in the National Pink Boots Society of female brewer “movers and shakers”. She co-runs the She-Brew fest that showcases women brewers and supports LGBTQ+ causes. Lee carved a niche for herself by creating dessert beer; today she has expanded her repertoire at Portland’s Ground Breaker Brewing. In recent years, Lee has also moved into distilling a full line of spirits including whiskey, rum and pear brandy at McMenamins Cornelius Pass Distillery. Along with a slew of homebrew projects, her most recent endeavor is the Barreled Bee, her own company that produces barrel-aged honey.

**PALLERONI, Sergio** (Portland State, Center for Public Interest Design) is a founder of Social Impact Design. He is a professor in Portland State’s School of Architecture, Director of the Center for Public Interest Design, a Senior Fellow of the Institute for Sustainable Solutions at the University and a founding member of the Green Building Research Lab. Twenty years ago, Sergio created the BaSiC Initiative, a service-learning fieldwork program which continues to move students out of the classroom and into the world to develop collaborative, sustainable projects with underserved communities. He has served as a consultant on sustainable architecture and development to international agencies, governments and organizations. In 2017, through the Center for Public Interest Design, he was a leader in a multi-sectorial collaborative process that resulted to the creation of Kenton Women’s Village in North Portland, a pilot project for a handful of women without homes that provides a transformative, sustainable response to the housing deficits in Portland.

————————————————————————

**(W-13) WEDNESDAY 8:00-9:50**

**Parlor A**

***Keeping Up with the Times: Negotiating the Nursing Profession in the 21st Century, Part I* (CONAA)**

What is it like to be a nurse in the 21st century? Ever-evolving and increasing technological advances create new opportunities and challenges to providing care. New approaches to healthcare are changing how it is provided and accessed. People are living longer but not necessarily healthier lives, shifting the types of care they need. Simultaneously, we face a global nursing shortage, adding pressure to the profession. Regardless, nurses remain at the center of healthcare delivery. This panel seeks to use an anthropological lens to explore the experience of the nursing profession today.

CHAIR: **BLUDAU, Heidi** (Monmouth U)

**JENNINGS, Bonnie** (School of Nursing, Emory U*) Using Ethnography to Understand Turbulence in Acute Care Settings* This study was conducted on a medical unit and a surgical unit to explore turbulence in contemporary hospitals. Nurses must navigate a complex mixture of demands within the temporal structure of their shifts to complete work that inherently entails interruptions. Unit clerks are the hub through which unit communication flows, either mitigating or magnifying turbulence. The findings call into question common conceptualizations of nurses’ work illustrating that tasks are rarely discrete but rather interwoven, inseparable, and best managed through articulation work. The findings also question the use of the “med-surg” nomenclature as it disguises important differences in these practice settings.

**BREDA, Karen** (U Hartford) and **PADILHA, Maria Itayra** (UFSC Federal U-Santa Catarina) *An Anthropological Critique of the Evolution of Health Care Providers in a Turbulent Health Care Market*. The fast-paced climate of today’s turbulent health care environment requires its service providers be agile and prepared to adapt and change. Uncertainty around acuity levels and the economics of health care reimbursement have pushed US corporate health care to increasingly incorporate advanced practice nurses (APNs) in the care provider mix. This paper will critique the reality of APNs in the US and analyze why the US market is particularly ripe for the use of this care provider. How this relates to the applied anthropology of health and what it means for applied anthropology research, practice and advocacy will be explored.

**PADILHA, Maria** (UFSC Federal U-Santa Catarina), **TOSO, Beatriz** (UNIOESTE-Cascavel-SC), and **BREDA**, Karen (U Hartford) *The Euphemism of ‘Good Nursing Practice’ or ‘Advanced Practice Nursing’* This paper reflects on an ongoing polemical debate through its discussion of good nursing practice in the patient care process as being central to nursing versus the development of advanced practice nursing (APN) in Brazil. It considers arguments for both subjects, grounding the discussion in the theoretical references of the two themes and proposes that, in addition to good nursing practice, undoubtedly necessary, the appropriation of advanced practice nursing and the debate about its adoption in Brazil is fundamental for the advancement of the profession and the formation of human resources for the unified health care system.

**BLUDAU, Heidi** (Monmouth U) *Handmaiden No More* This paper discusses migration of Czech nurses to the Middle East and their subsequent return. An impetus for this migration is a search for professional respect. Nurses in the Czech Republic are often still the physician’s assistant rather than an autonomous practitioner. Ethnographic data is used to examine how nurses seek and negotiate increased responsibilities in foreign hospitals and the ways in which nurses address returning to the Czech environment. A key element is corresponding care ideologies between the nurse and the work environment, including the marked difference found in this study between labor and delivery and other areas of care.

**ENGEBRETSON, Joan** (U Texas) *Moving from the Industrial Age to the Information Age: Implications for Nursing Science* The cultural aspects of society and medicine have been highly influenced by the Industrial age with a focus on mechanical science and efficiency. This is reflected in healthcare by the centrality of evidence-based practice, cost effectiveness and specialization. As we move into the information age, newer approaches focus on biological systems as complex adaptive systems. This move toward systems thinking has important implications for applied anthropology as well as for nurses, who have long focused on a better understanding of these complex landscapes of embedded systems.

———————————————————————––

**(W-43) WEDNESDAY 10:00-11:50**

**Parlor A *Keeping Up with the Times: Negotiating the Nursing Profession in the 21st Century, Part II (CONAA)***

CHAIR: **BLUDAU, Heidi** (Monmouth U)

**ANDERSON, Barbara** (Frontier Nursing U) *The U.S. Nursing Shortage: Determinant of National and Global Health* The nursing shortage is a global problem. In America, the numbers of graduates have not met the growing health care needs of the nation. Downsized university budgets restrict adequate numbers of nursing faculty and clinical sites. The shortage affects rural and frontier areas in the U.S. where many critical access hospitals have been closed. The U.S. nursing shortage also impacts low-resource nations who experience large-scale migration of nurses helping to fill our gap but resulting in closure of health services and public health programs in poor nations. The U.S. needs a formalized national policy and immediate action to develop a sustainable, domestic workforce.

**SELLERS, Kathleen F.** (SUNY Polytechnic) *Rural Nursing Retention* Rurality is a multidimensional construct including ecological, occupational and sociocultural frameworks (Bealer, et al.,1965). Rural nursing practice has repeatedly been found to have unique challenges that impact retention. Nurses more often choose a job in a rural area if they are connected to and trained at rural facilities and if they perceive rural workplaces to be supportive (Bushy et al., 2005). Roberge (2009) reported that nurses were only satisfied with their jobs if they were also satisfied with their community. This current study found that the fit between the nurse and the community plays a key role in understanding rural nurse engagement and retention.

**EMERSON, Christie** (Kennesaw State U) *I Was Clear with My Goals, Where I’m Heading, and What I Wanted with My Life: Life History of an Omani Woman and Nurse Leader* This research documents the life history of an Omani woman who grew up during the Omani renaissance, chose a career in nursing, and subsequently became a nurse leader in Oman. Life history methodology was used to elicit rich descriptions of the context, thoughts, and experiences that the key participant chose to use in telling the story of her life. Themes and subthemes that emerged from her story were: 1) opportunity, with subthemes of national identity, country building, and nursing pioneer; 2) visionary, with subthemes of leadership, perseverance, resilience, and mentors; and 3) nursing, with subthemes of advocacy, caring, and fulfillment.

**KUERTEN ROCHA, Patricia**, **DASILVA, Maria**, **PADILHA, Maria** (UFSC Federal U-Santa Catarina), **BIAZUS DALCIN, Camila** and **ANDERS, J.C.** (UFSC Federal U-Santa Catarina) *Construction of an Instrument for Handoff in Brazilian Pediatric Hospital Units* Effective healthcare communication is a universal goal for patient safety. Culture and social structure can influence communication errors among health care professionals. Few tools exist to assess the quality of handoff between nurses and physicians. This paper reports on the validity of a handoff tool tested for use in Brazil in pediatric settings. Methodological research for construction and content validation of the instrument was based on a literature review of the SBAR (Situation, Background, Assessment and Recommendation) tool. It is possible to standardize the information and optimize communication for pediatric patient safety and to promote inter-disciplinary practice between nurses and physicians.

———————————————————————––

**(W-73) WEDNESDAY 12:00-1:20**

**Parlor A**

***Evidence and Advocacy for Safe, Accessible Health Care (CONAA)***

By examining programs for childbearing women, children with complex needs, people living with diabetes, and health profession students, the panelists address this question: How can we improve health programming and service delivery to improve patient safety and well-being for vulnerable populations? Through program development and evaluation, phenomenology of service experiences, and analysis of health systems, social and political priorities, we discuss how to promote health and advocate for accessible services. Together, these papers explore current health status and services for diverse populations and suggest changes at training, intervention, system, and societal levels.

CHAIR: **MATTHEWS, Elise** (U Regina)

**ANDERSON, Barbara** (Frontier Nursing U) *Where is my Mama?: Escalating Maternal Mortality in America* Maternal mortality in the United States (death during pregnancy or the first year postpartum) is the highest among all developed nations, rising exponentially in the last 15 years. The greatest impact is on African-American and American Indian/Alaska Native women. Cardiovascular events have replaced hemorrhage as the leading cause of death. Severe morbidity is also high with many “near-miss” events. This crisis is grounded in the social determinants of health, structural-systemic forces, declining accessibility to care, insufficient number of maternal health providers, and a lack of national prioritization of maternal health. This presentation engages participants in interactive critical thinking about advocacy across multiple sectors.

**KONZELMAN, Gregory** (CONAA) *The Art of Communication in a Primary Care Setting* Clear and accurate communication is the key in human relationships. This is particularly relevant in the clinical nursing setting. Inaccuracy in either patient assessment and/or communication between the RN and the health provider can be disastrous. This presentation focuses on accurate assessment and clear communication with the provider. A triage lecture series was developed to have nurse practitioners give presentations on specific body systems and potential “red-flag” findings. As the series continues to develop and improve, its goal is to minimize missed findings and improve patient safety.

**CHANDLER, Chelsea**, **WHOLLEY, Samantha**, and **CROCKER, Theresa** (USF) *Engaging Change in the Trying and Turbulent Times of Completing a Dietetic Internship* This research stems from our experience as dietetic interns in the newly implemented University of South Florida Dietetic Internship. This necessary portion of the journey to become a professional Registered Dietitian (RDN) tests the limits of even the most academically successful students as participants are tested mentally, physically and emotionally. The incorporation of relaxation and stress management techniques by medical students has been shown to result in less burnout, discomfort, and anxiety. Considering the rigorous nature of the integrated program, we explore the feasibility of incorporating stress management techniques to benefit future populations and cultivate successful Registered Dietitians.

**SHEEHAN, Lisa** (USD) and **BURSCH, Lisa** (CA Baptist U*) Improving Provider Diabetes Care in a Student-Run Free Clinic* The purpose of the free clinic is to improve outcomes in vulnerable patients with diabetes. It is designed to improve diabetes guideline knowledge utilization and electronic medical record (EMR) documentation adherence rates of Nurse Practitioner (NP) student providers in student run free health care clinics. NP student provider education related to American Diabetes Association recommendations and guidelines resulted in decreased A1c in patients, suggesting that provider diabetes care guideline and EMR documentation rate adherence improves outcomes for patients with diabetes.

**MATTHEWS, Elise** and **PUPLAMPU, Vivian** (U Regina) *Strategies of Adaptation among Parents of Children with Neurodevelopmental Disorder* Parents of children with neurodevelopmental disorders experience challenges in a context of budgetary fiscal austerity in disability, health, education, and social services. Parents work at navigation, peer-referral and advocacy for their children with diverse support and service needs, including those related to a high rate of co-occurring mental health disorders (e.g., depression, anxiety, psychosis). Interviews were conducted with 40 parents of 47 children with neurodevelopmental disorders (e.g., Autism) across urban and rural Saskatchewan. This research revealed many strategies of adaptation by parents facing difficulties accessing services, which could guide changes in health systems.

———————————————————————––

**(TH-103) THURSDAY 1:30-3:20**

**Parlor A**

***Resilience and Change in the Chaos of War, the Uncertainty of Urban Landscape, and the Upheaval of Healthcare (CONAA)***

Change is part of life and often occurs in turbulent times. How do individuals and populations manage change and turbulence? In this session the presenters will describe how individuals, families, and communities engaged with changes during turbulent times and created positive outcomes. The audience will learn about the changes made to deal with both urban renewal and urban sprawl; changes in healthcare delivery to serve the needy; and changes made by families escaping war and genocide to live in the US. These presentations will serve as reminders of human resilience and adaptability when people are faced with change.

CHAIR: **BROWN, Brenda** (Kennesaw State U)

**JALIL-GUTIERREZ, Sylvia** (CCSU Central CT State U) *Change. Displacement, and Resilience in the Face of Economic Collapse: A Case Study of a Mid-sized New England Town* This paper is a critical analysis of urban renewal in a New England town. How do the residents of this mid-sized town make sense of the changes that occurred through urban renewal? How did quality of life change? Using archival research, interviews and participant-observation, the history of urban renewal is documented from the 1950s to the present. Through community voices, changes in the urban landscape and the impact on the health and well-being of community residents are explored along with how the changes were (and are) contested and rejected. Essential to this exploration is an examination of how class, race and gender are affected by urban redevelopment.

**NORRIS, Susan** (Immaculata U) *Changing (Dis)Course: Using the Intersection of Perspectives and Practice to Understand the Health Needs of an Urban Community* Encouraging local experts to collectively engage with each other about the health of a community by sharing knowledge, experiences, and perspectives can create meaningful engagement that fosters the sense of community, helps to identify local development initiatives promoting change, and find intersecting points to potentially improve health outcomes. This paper is a thematic analysis of a critical discussion that brought together thought leaders in Philadelphia to focus on the health of urban dwellers. Through a lens of sustainability in the face of change, the intersection of the quality of air, water, food in the context of poverty are considered with an emphasis on their impact on the health of vulnerable populations.

**DZUBUR, Valerie** (Samuel Merritt U) *Human Migration in the Context of War and Genocide: Lessons Learned from the Bosnian Experience Where “They Killed Our Lives”*This presentation will present a story of human migration in the context of war and ethnic cleansing. We know that human development is disrupted in children, identities are forfeited and cultural norms are ruptured. More specifically this discussion uses the Bosnian experience of four families that escaped to the United States in 1995.These families escaped the siege of Sarajevo by crawling through the now-famous tunnel, constructed under the city, to reach the airport. Now twenty years later it is informative to consider the process of healing and rebuilding that underpinned the recovery of their lives.

**BROWN, Brenda** (Kennesaw State U) *Changing Healthcare Delivery to Meet the Needs of Refugees: The Story of the Clarkston Clinic* As refugee numbers soar so does the need for culturally sensitive, affordable, and accessible healthcare. Clarkston, GA is home to a large and diverse refugee population. The author will present the story of one clinic which began as a mobile unit and is now a freestanding building located in the heart of Atlanta’s refugee population. The physicians, nurses, students, and other volunteers who staff the clinic are grateful for the opportunity to serve the community. During these turbulent times in the US for refugees and immigrants, the clinic and staff are willing to make changes so that all may benefit.

**SHAVER, Amy** (Utica College) and **SELLER, Kathleen** (SUNY Poly) *Rural Elders’ Experiences and Insights into Their Changing Community* This phenomenological anthropological study explored the lived experience of suburban sprawl for rural elders. Their stories of change lend insight into effects of this phenomenon on elders aging in place and on the deeply rooted rural culture of the community. Both etic and emic approaches were taken as researchers became part of the lives of elders in a small community in New York State that has been part of urban-rural migration. Outcomes of the study shed light on positive and negative changes and the elders’ experience of adapting to the change while sustaining their rich heritage

———————————————————————––

**(TH-133) THURSDAY 3:30-5:20**

**Parlor A**

***Exploring Change among the Vulnerable: Interdisciplinary Perspectives (CONAA)***

Drawing on this year’s theme of engaging change in turbulent times, this session brings together five papers that explore the notion of change and its impact on vulnerable populations across the lifespan. Presenters will draw on current research and theoretical approaches from applied anthropology, occupational therapy/occupational science, disability studies, and nursing to explore the concept of embodied knowledge and how change is perceived, embraced or resisted based on positionality and access to opportunities, resources, etc. Using their findings from case studies, ethnographies, and program development projects, this session will advance this ongoing discussion.

CHAIR: **PAUL-WARD**, Amy (FIU)

**PAUL-WARD, Amy** (FIU) *Addressing Instability, Transition, and Change for Emerging Adults in Foster Care* The transition from foster care to independent living is one of immense change and can be fraught with instability. While this instability stems from many factors, it is compounded by the fact that many of these young people have not had the opportunity to develop the skills necessary for independent adulthood. Drawing from an ongoing program development project, multiple theoretical perspectives will utilize applied anthropology, occupational justice and disability studies to explore the notion of change, what it means, and how it is experienced by a group of vulnerable emerging adults preparing to leave foster care.

**KABEL, Allison** (Towson U) *Clothing, Participation & Masculinity: Case Studies on Apparel Function and Disability* Two case studies were collected through collaboration with men living with long-term, compromised spinal conditions. They described the relevance of clothing and apparel to their overall wellbeing, safety and personal style. Participants described clothing that failed to meet needs for durability, comfort, and safety. Apparel choices required planning to prevent injury while still meeting standards of social acceptability. Temperature regulation was a particular concern when selecting clothing. Participants reported skepticism about adaptive apparel for people who use wheelchairs. Narratives and observations support the concept of social participation as an accomplishment, with apparel playing a vital role in achieving and maintaining participation.

**KENDRICK, Lorna** (Samuel Merritt U) and MOORE, Lorraine (Life West Chiropractic U) *Using Mindfulness to Engage Change in the Physical and Mental Health of Disparaged Groups in Turbulent Times* Societal stressors on people of color and other disparaged groups increases their risks for physical and mental health disparities. Moreover, with the current turbulent political climate there are increased tensions and strains among these groups that will have long-lasting effects on mental and physical health for years to come. This collection of case studies focuses on ways mindfulness can be used to promote an individual’s awareness of and reaction to their body’s responses to environmental factors.

**SHAY, Kimberly** (Wayne State U) *Change and Continuity in Older Age: Maintaining Personhood among Aging Museum Volunteers* For many adults, older age is a time of transition and change, in both physical and cognitive abilities. These changes are frequently difficult and often inevitable, however, the ability to mitigate those changes and support individuals as they strive to maintain their prescribed roles and maintain their full personhood in the communities in which they are engrained, must be a collective group effort. Using examples from an ongoing ethnographic project and drawing on the insight from a cohort of aging museum volunteers, the ways in which change is experienced, embraced and resisted, by older adults in this museum is examined.

**CARRILLO, Erika** (Purdue U) *Accommodating Meal Time: The Central Role of Food in Elder Caregiving Discussions among San Francisco Families* In this presentation, forms of caregiving in a local senior center, homes, and other places of social significance in San Francisco, California’s Mission District are examined. From the perspectives of applied anthropology and gerontology, key cases from a larger, focused research project will be presented. How caregivers for older Latinos define, enact, and negotiate “good” care in the rapidly transforming Mission District will be discussed and through the utilization of ethnographic methods, forms of caregiving for Latino seniors and the discussions that surround food, nutrition, and accommodations during meal time during important life transitions that some seniors experience in later life are analyzed.

**Special CONAA Event**

**(TH-171) THURSDAY 5:30-7:00**

**Senate Suite**

**CONAA Business Meeting 5:30 to 7:00pm followed by CONAA dinner in local restaurant**

